# Progressive Multifocal Leukoencephalopathy as the Initial Presentation of Undiagnosed HIV Infection: A Case Report

**DOI:** 10.7759/cureus.93923

**Published:** 2025-10-06

**Authors:** Abdul Haseeb, Qazi Tauseef Ahmad, Abdul Basit, Muzzamil Samad, Ammara Afridi

**Affiliations:** 1 Internal Medicine, Lady Reading Hospital, Peshawar, PAK; 2 Internal Medicine, Khyber Teaching Hospital, Peshawar, PAK; 3 Internal Medicine, Khyber Medical College, Peshawar, PAK

**Keywords:** acquired immune deficiency syndrome (aids), highly active antiretroviral therapy [haart], hiv aids, john cunningham (jc) virus, progressive multifocal leukoencephalopathy (pml)

## Abstract

Progressive multifocal leukoencephalopathy (PML) is a rare and often fatal complication associated with advanced HIV infection, caused by the reactivation of the John Cunningham (JC) virus in immunocompromised individuals. Diagnosis is challenging due to nonspecific symptoms and the need for specialized diagnostic tools, which may not be readily available in resource-limited settings. Early initiation of antiretroviral therapy (ART) is critical for managing the condition, though prognosis remains poor in advanced cases.

We present the case of a 30-year-old male with newly diagnosed HIV who presented with a two-week history of fever, headache, hiccups, and unconsciousness. Initially suspected to have encephalitis, the patient was empirically treated with ceftriaxone, acyclovir, and later vancomycin. HIV testing, performed with an independent kit, confirmed a positive diagnosis, and ART was started. A CSF analysis revealed findings suggestive of tuberculous meningitis, and treatment for tuberculosis was initiated. However, subsequent re-evaluation of MRI images raised suspicion of PML, which was confirmed radiologically. Due to resource constraints, JC virus polymerase chain reaction testing was not conducted, and further diagnostic tests were unavailable. The patient’s condition continued to deteriorate despite supportive care, and he succumbed to complications of immunosuppression on the 13th day of admission.

This case highlights the diagnostic challenges of PML in patients with HIV, especially in settings with limited resources. The patient's initial presentation with nonspecific neurological symptoms and delayed diagnosis of HIV and PML contributed to poor outcomes. Early ART initiation remains essential, though its efficacy may be limited in cases of severe immunosuppression.

PML should be considered in the differential diagnosis of neurological symptoms in patients with newly diagnosed or advanced HIV. Early ART initiation is crucial, though the prognosis remains poor in severe cases. This case emphasizes the importance of comprehensive neurological assessment and vigilance in individuals with HIV presenting with new-onset neurological symptoms.

## Introduction

Progressive multifocal leukoencephalopathy (PML) represents one of the most devastating opportunistic infections affecting the central nervous system in immunocompromised patients. First described by Åström et al. in 1958, PML is caused by reactivation of the ubiquitous John Cunningham (JC) virus, a double-stranded DNA polyomavirus that remains latent in the majority of the adult population [1]. Under normal immunological conditions, JC virus remains dormant; however, severe immunosuppression permits viral reactivation, leading to productive infection of oligodendrocytes and subsequent demyelination [2].

The epidemiology of PML has evolved significantly since the advent of the HIV pandemic. While historically associated with hematological malignancies and chronic immunosuppressive therapy, approximately 80%-85% of PML cases now occur in the context of HIV infection globally [3]. The incidence of PML in HIV-positive patients ranges from 0.07% to 5%, with the highest risk occurring when CD4+ T-cell counts fall below 200 cells/μL [4]. Despite the widespread implementation of highly active antiretroviral therapy (ART), PML continues to carry a grave prognosis, with mortality rates exceeding 50% even with optimal treatment [5].

The clinical presentation of PML is characterized by progressive, multifocal neurological deficits that reflect the anatomical distribution of white matter lesions. Common manifestations include hemiparesis, gait ataxia, visual field defects, cognitive impairment, and speech disturbances [6]. The diagnosis relies on a combination of clinical presentation, characteristic neuroimaging findings, and demonstration of JC virus DNA in CSF through polymerase chain reaction (PCR) testing [7].

While PML typically occurs in patients with established HIV infection and known immunosuppression, rare cases have been reported as the initial manifestation of undiagnosed HIV disease [8]. These cases present unique diagnostic challenges, as the underlying immunodeficiency may not be immediately apparent, leading to delays in appropriate diagnosis and treatment. This case report describes a young male who presented with acute neurological deterioration secondary to PML, ultimately leading to the diagnosis of previously unrecognized HIV infection.

## Case presentation

A 30-year-old previously healthy male presented to the ED with a two-week history of persistent high-grade fever, severe headache, intractable hiccups for one week, and an acute onset of altered consciousness over the preceding 24 hours. The patient had no known medical history, was not taking any medications, and denied any high-risk behaviors for HIV transmission when directly questioned. Family history was unremarkable for immunodeficiency disorders or malignancies.

On initial presentation, the patient appeared acutely ill with a Glasgow Coma Scale (GCS) score of 9/15 (E3M4V2). Vital signs revealed: blood pressure: 110/80 mmHg, heart rate: 97 beats per minute, respiratory rate: 18 breaths per minute, temperature: 38.5°C, and oxygen saturation: 99% on room air. Physical examination was notable for the absence of neck stiffness, lymphadenopathy, or skin lesions. Neurological examination revealed an altered mental status with inappropriate responses to verbal commands, but no focal neurological deficits were initially apparent.

Given the clinical presentation of fever, headache, and altered consciousness, viral encephalitis was suspected as the primary diagnosis. Empirical antimicrobial therapy was immediately initiated with intravenous ceftriaxone 2 g every 12 hours and acyclovir 10 mg/kg every 8 hours.

Initial laboratory results revealed several abnormalities as detailed in Table 1. Notable findings included microcytic anemia (hemoglobin: 10.3 g/dL and mean corpuscular volume: 67.9 fL), mild hyponatremia (sodium: 132 mmol/L), and otherwise normal basic metabolic panel, liver function tests, and coagulation studies.

**Table 1:** Laboratory investigation along with results in normal ranges ALT, Alanine aminotransferase; Anti-HCV, Antibody to hepatitis C virus; Anti-HIV, Antibody to human immunodeficiency virus; GPT, Glutamate pyruvate transaminase; HBSAg, Hepatitis B surface antigen; HCT, Hematocrit; HGB, Hemoglobin; ICT, Immunochromatographic test; MCH, Mean corpuscular hemoglobin; MCHC, Mean corpuscular hemoglobin concentration; MCV, Mean corpuscular volume; PLT, Platelet count; RBC, Red blood cell; WBC, White blood cell; WHO, World Health Organization.

Test	Result	Normal range
WBC	7.76 x 10^3^/µL	4-11 × 10^3^/µL
RBC	4.46 x 10^6^/µL	4-6 x 10^6^/µL
HGB	10.3 g/dL	11.5-17.5 g/dL
HCT	30.3%	35.0%-46.0%
MCV	67.9 fL	76-96 fL
MCH	23 pg	27-33 pg
MCHC	33.9 g/dL	33-35 g/dL
PLT	155 x 10^3^/µL	150-450 x 10^3^/µL
Sodium	132 mmol/L	135-150 mmol/L
Potassium	3.73 mmol/L	3.5-5.1 mmol/L
Chloride	99 mmol/L	96-112 mmol/L
Blood urea	28.46 mg/dL	18-45 mg/dL
Creatinine	0.94 mg/dL	0.64-1.2 mg/dL
Total bilirubin	0.2 mg/dL	0.1-1.0 mg/dL
ALT/GPT	13 U/L	10-50 U/L
Alkaline phosphatase	51 U/L	40-129 U/L
HBSAg (by ICT)	Negative	
Anti-HCV (by ICT)	Negative	
Anti-HIV (by ICT)	Negative	
Anti-HIV (WHO HIV Early Detect kit)	Positive	

Despite six days of empirical antimicrobial therapy, the patient showed no clinical improvement. His neurological status progressively deteriorated, with GCS declining to 7/15 by hospital day 6. Neurological examination at this time revealed asymmetric pupils (right pupil dilated and nonreactive to light, and left pupil mid-dilated with sluggish light response), motor asymmetry (right upper extremity demonstrating flexion response to painful stimuli and left upper extremity showing no response), and asymmetric plantar reflexes (right downgoing and left equivocal). Given the lack of response to initial therapy, vancomycin 1 g every 12 hours was added to the regimen.

During this period, a discrepancy in HIV testing results emerged. The routine hospital HIV immunochromatographic test (ICT) had returned negative; however, the hospital's WHO-supported HIV testing center performed confirmatory testing using the HIV Early Detect Kit by Abbott, which yielded a positive result. This discrepancy may reflect the seroconversion window period, variability in sensitivity between rapid kits, or potential operator-related error. Given the positive HIV result, combination ART was immediately initiated with a once-daily regimen of dolutegravir/lamivudine/tenofovir disoproxil fumarate.

The CSF findings (Table 2) demonstrated elevated protein (188 mg/dL), markedly decreased glucose (15 mg/dL), lymphocytic pleocytosis, and absence of bacterial organisms on Gram staining. These findings were highly suggestive of tuberculous meningitis. Consequently, anti-tuberculosis (TB) therapy was immediately initiated with the standard four-drug regimen: isoniazid, rifampin, ethambutol, and pyrazinamide, along with pyridoxine (vitamin B6) supplementation. Although anti-tuberculous therapy was started empirically based on CSF analysis report, CSF adenosine deaminase and TB-PCR were not available due to financial restrains.

**Table 2:** CSF analysis results CSF, Cerebrospinal fluid

Parameter	Result	Units	Normal range
Volume	2	mL	-
Appearance	Clear, colorless	-	Clear, colorless
Protein	188	mg/dL	15-60 mg/dL
Glucose	15	mg/dL	50-80 mg/dL
White blood cell count	18	cells/mm³	0-5 cells/mm³
Red blood cell count	270	cells/mm³	0-10 cells/mm³
Neutrophils	3	%	0%-6%
Lymphocytes	97	%	40%-80%
Gram stain	No organisms seen	-	No organisms
Acid-fast bacilli stain	No acid-fast bacilli seen	-	No acid-fast bacilli

On hospital day 9, the radiology department conducted a comprehensive re-evaluation of the patient's brain MRI studies, taking into consideration his newly diagnosed HIV status. This review raised suspicion for PML rather than the initially suspected infectious etiologies. A formal re-report issued on hospital day 10 confirmed the radiological diagnosis of PML based on characteristic imaging features and clinical context.

**Figure 1:**
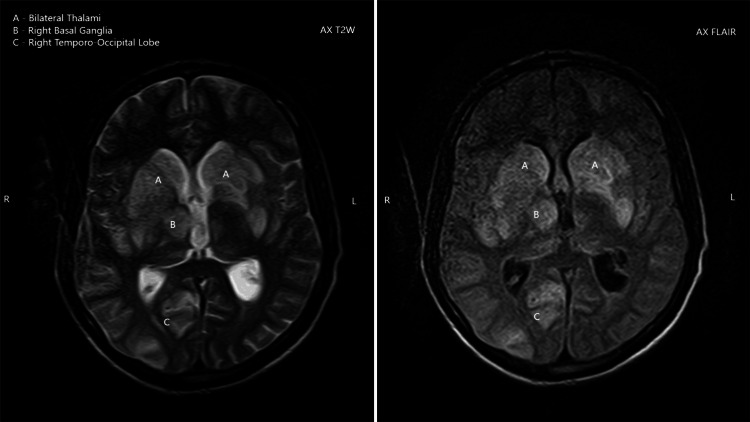
Axial T2-weighted and axial FLAIR images showing hyperintense lesions in the bilateral thalami, right basal ganglia, and right temporo-occipital white matter, with no significant mass effect, a pattern consistent with progressive multifocal leukoencephalopathy

**Figure 2:**
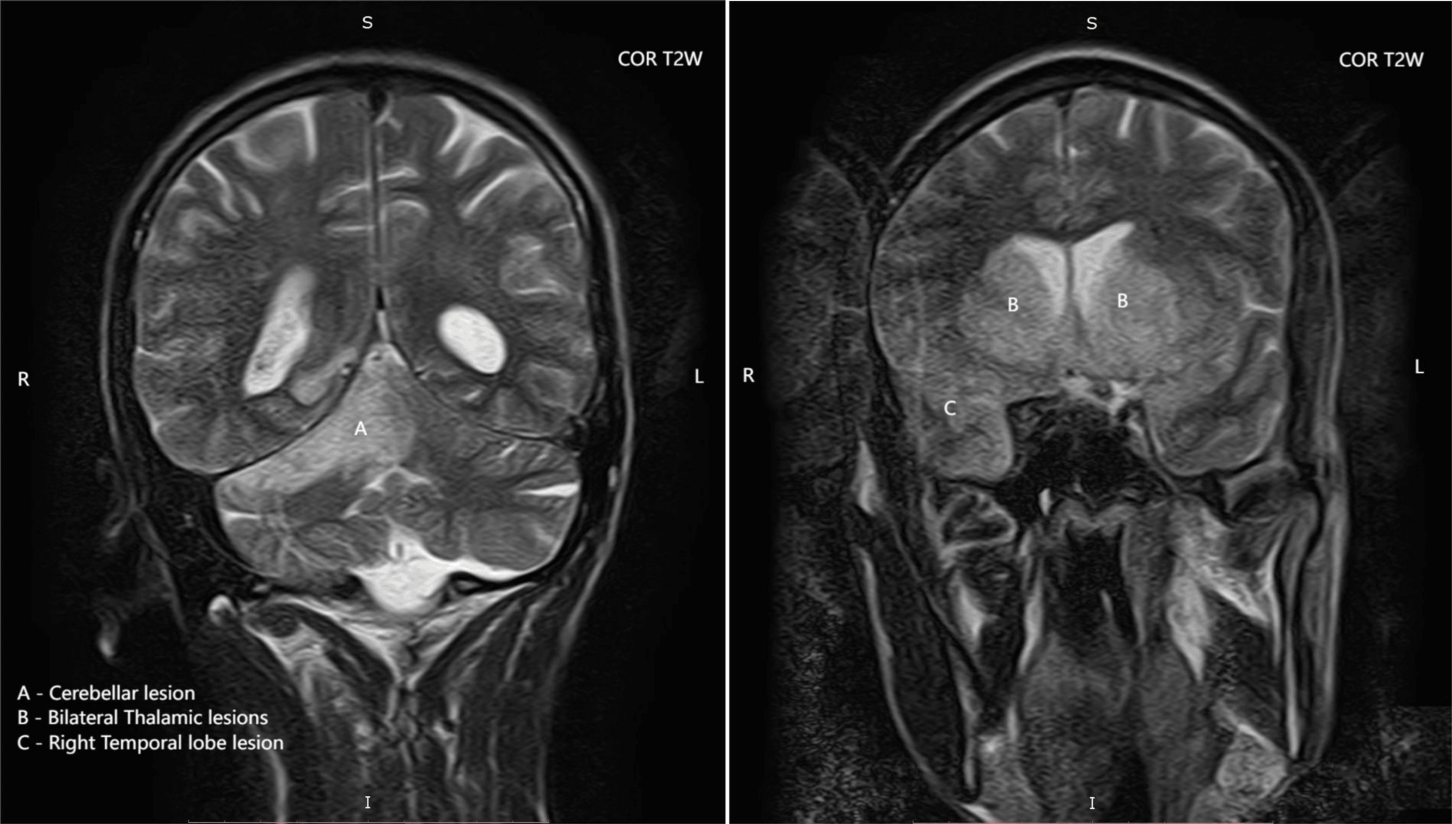
Coronal T2-weighted MRI demonstrating high signal intensity lesions involving (A) the cerebellum, (B) the bilateral thalami, and (C) the right temporal lobe MRI, Magnetic resonance imaging

**Figure 3:**
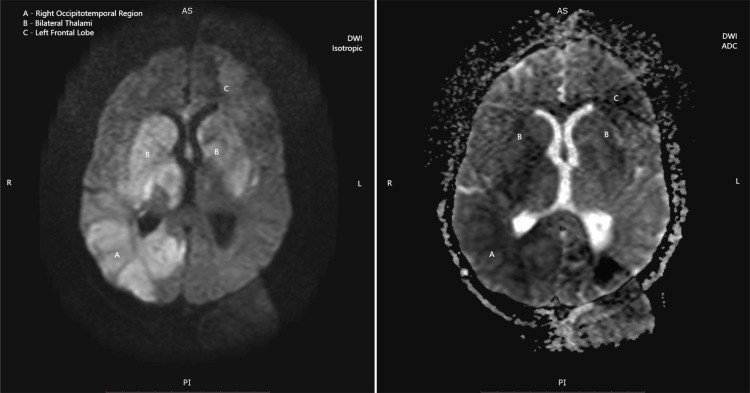
Diffusion-weighted MRI (DWI isotropic, left) and corresponding ADC map (right) highlighting multifocal areas of diffusion restriction ADC, Apparent diffusion coefficient; DWI, Diffusion-weighted imaging; MRI, Magnetic resonance imaging

**Figure 4:**
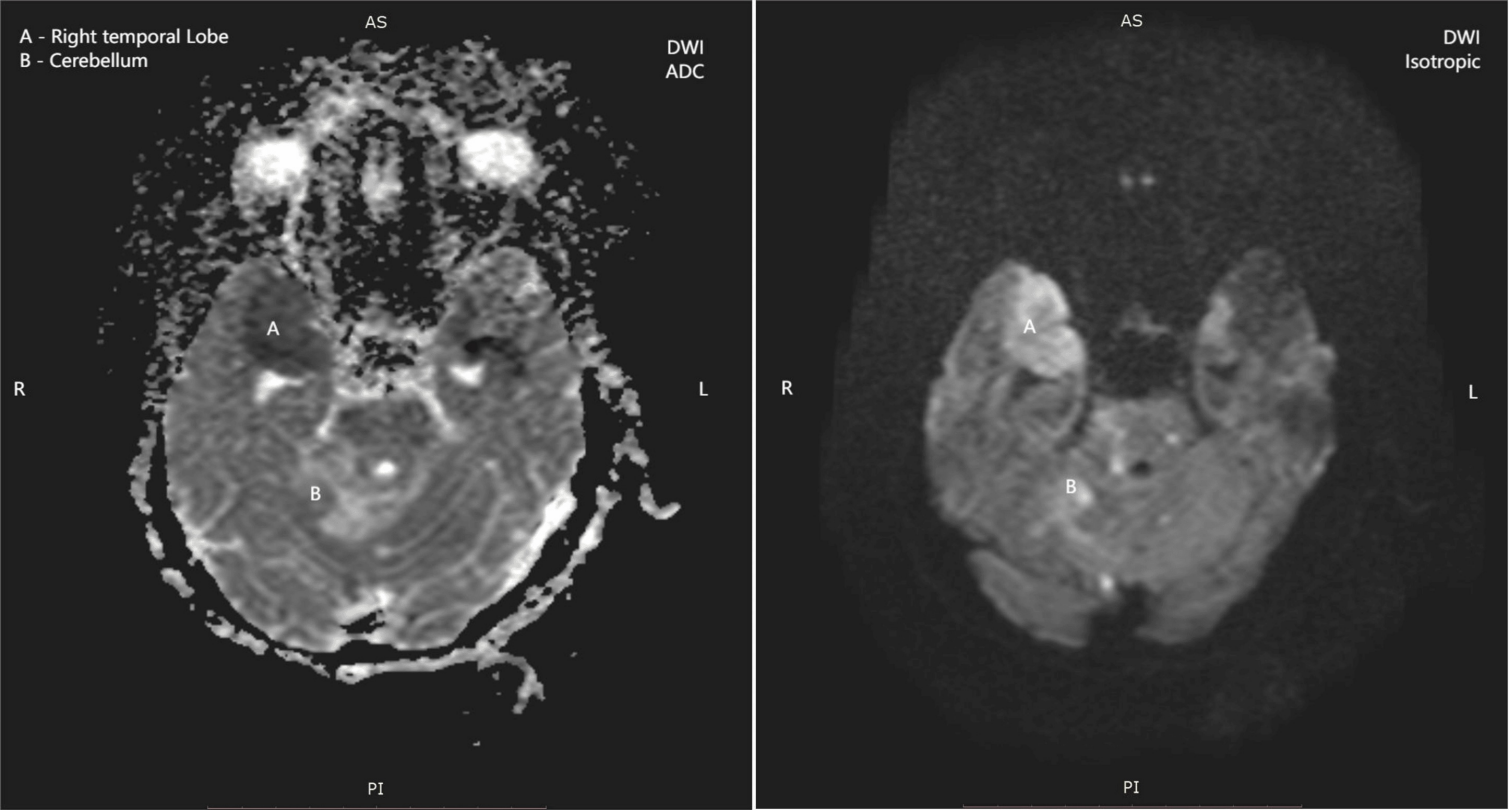
Axial DWI and corresponding ADC map showing diffusion restriction in the right temporal white matter and cerebellar hemisphere ADC, Apparent diffusion coefficient; DWI, Diffusion-weighted imaging

The MRI demonstrated multifocal, asymmetric white matter lesions with several pathognomonic features of PML: (1) T1-weighted images: Lesions appeared isointense to hypointense relative to normal white matter. (2) T2-weighted and FLAIR images: Hyperintense signals were observed in the bilateral thalami, right basal ganglia, and right temporo-occipital lobe (Figure 1), and coronal T2-weighted images showing hyperintense signals in the bilateral thalami, right temporal lobe, and cerebellum (Figure 2). (3) Diffusion-weighted imaging (DWI): True diffusion restriction was evident in the bilateral basal ganglia, right thalamus, parietal lobe, and right temporal lobe (Figures 3, 4). (4) Distribution pattern: The lesions showed characteristic involvement of subcortical U-fibers and deep white matter structures.

In the absence of JC virus PCR testing, and given the patient’s financial constraints and guardians’ reluctance for invasive procedures, the diagnosis remained presumptive. However, the imaging features in the clinical context of advanced HIV were strongly supportive of PML.

To provide greater clarity, the sequence of symptoms, investigations, and interventions is summarized in Table 3. This timeline highlights the rapid progression and diagnostic challenges encountered during the patient’s hospital course.

**Table 3:** Timeline of clinical course, investigations, and management 3TC, Lamivudine; ART, Antiretroviral therapy; CBC, Complete blood count; CSF, Cerebrospinal fluid; DTG, Dolutegravir; GCS, Glasgow Coma Scale; HIV, Human immunodeficiency virus; ICT, Immunochromatographic test; LFT, Liver function test; MRI, Magnetic resonance imaging; PML, Progressive multifocal leukoencephalopathy; TB, Tuberculosis; TDF, Tenofovir disoproxil fumarate; WHO, World Health Organization

Day (relative to admission)	Clinical event/findings	Investigations	Management/interventions
-14 to -1	High-grade fever, severe headache, intractable hiccups	–	–
0 (admission)	Altered consciousness (GCS 9/15), fever, no meningeal signs	Initial labs (CBC, electrolytes, LFTs, renal profile); baseline ICT for HIV negative	Empirical ceftriaxone + acyclovir started
1–5	Persistent fever, no improvement; progressive neurological decline	–	Supportive care continued
6	Further deterioration (GCS 7/15, asymmetric pupils, motor asymmetry)	–	Vancomycin added
6–7	HIV testing discrepancy noted: ICT negative, confirmatory WHO HIV Early Detect kit positive	Confirmatory HIV positive	Initiation of ART (DTG/3TC/TDF)
7	Lumbar puncture performed	CSF: protein ↑, glucose ↓, lymphocytic pleocytosis, no organisms; suggestive of TB meningitis	Empirical anti-TB therapy started
9–10	Radiology re-evaluation in light of HIV positivity	MRI: multifocal asymmetric white matter lesions consistent with PML	Diagnosis revised to presumptive PML
11–12	Continued neurological decline	–	Empirical antifungal therapy (amphotericin B, fluconazole) initiated for possible cryptococcosis
13	Progressive deterioration, multifocal deficits	–	Supportive care; the patient succumbed to complications

Unfortunately, definitive diagnostic confirmation through JC virus PCR testing of CSF was not feasible due to financial limitations and institutional constraints. Given the patient's severely immunocompromised state and the possibility of concurrent opportunistic infections, empirical antifungal therapy was added, including intravenous amphotericin B (3 mg/kg daily) and fluconazole (800 mg daily).

The patient's condition continued to deteriorate despite aggressive supportive care and targeted therapies. Progressive neurological decline was evident, with further reduction in consciousness level and development of additional focal neurological deficits. After 13 days of intensive care management, the patient succumbed to complications related to severe immunosuppression and PML.

## Discussion

Our case underscores the difficulty of establishing a definitive diagnosis of PML in low-resource settings. While JC virus PCR is the gold standard, this was not feasible in our setting. Hence, the diagnosis of PML was presumptive, based on classical MRI features in the appropriate clinical context.

The initial presentation of our patient with fever, headache, and altered consciousness is consistent with various central nervous system infections, making early differentiation challenging [9]. The nonspecific nature of these symptoms, combined with the absence of known HIV risk factors, initially directed clinical suspicion toward more common causes of viral encephalitis. This diagnostic uncertainty was compounded by the false-negative result of the initial HIV screening test, emphasizing the importance of using highly sensitive testing methods.

The discrepancy between routine hospital HIV testing and the WHO-supported confirmatory test highlights a critical issue in HIV diagnosis, particularly in resource-limited settings [10]. In our case, the initial negative ICT result may have reflected the seroconversion window period, reduced sensitivity of the rapid kit used, or even operator-related variability. Such discrepancies underscore the importance of confirmatory testing whenever clinical suspicion for HIV remains high, especially in patients presenting with unexplained opportunistic infections.

The radiological features observed in our patient were characteristic of PML, demonstrating the classical pattern of multifocal, asymmetric white matter lesions with predilection for subcortical regions [11]. The presence of diffusion restriction on DWI, while not universally present in PML, has been increasingly recognized as a potential finding, particularly in acute phases of the disease [12].

The CSF findings in our patient presented a diagnostic dilemma, as the elevated protein, low glucose, and lymphocytic pleocytosis were more suggestive of tuberculous meningitis than typical PML [13]. Classical PML cases often demonstrate normal or only mildly abnormal CSF parameters, with the primary diagnostic value of lumbar puncture being the detection of JC virus DNA through PCR testing [14].

The management of PML remains largely supportive, with ART being the primary intervention for HIV-associated cases [15]. The goal of ART is to restore immune function sufficiently to control JC virus replication; however, the effectiveness of this approach is significantly limited by the degree of immunosuppression and extent of neurological damage [16].

This case highlights significant challenges faced in resource-limited healthcare settings, where advanced diagnostic modalities may not be readily available. The inability to perform JC virus PCR testing, CD4+ cell count determination, and HIV viral load measurement significantly impacted both diagnostic certainty and prognostic assessment.

## Conclusions

PML should be considered in the differential diagnosis of neurological symptoms in patients with advanced HIV infection, especially those with newly diagnosed or poorly controlled HIV. Early recognition and initiation of ART are essential for improving patient outcomes, although the prognosis remains poor in severe cases. This case underscores the importance of comprehensive neurological assessment and heightened vigilance in individuals with HIV, particularly those presenting with new-onset neurological symptoms. Healthcare providers must remain vigilant for opportunistic infections and ensure access to appropriate diagnostic and therapeutic resources.
